# Cartas al Editor

**DOI:** 10.7705/biomedica.7554

**Published:** 2024-05-30

Cartas al editor

Lima, 8 de noviembre de 2023

Señores

Comité Editorial

Revista *Biomédica*

Bogotá, D. C., Colombia

Estimados editores:

Hemos llevado a cabo una revisión cuidadosa del artículo titulado “Colestasis intrahepática por *Treponema pallidum* en paciente inmunocompetente” de Orozco-Sebá y colaboradores (2023). El artículo presenta un caso de hepatopatía secundaria a sífilis y destaca la relevancia de las intervenciones y medidas no farmacológicas para reducir la morbimortalidad asociada con enfermedades de transmisión sexual, entre ellas, la recomendación de aplicar la vacuna contra el virus de la hepatitis C (HCV) ^1^.

En este contexto, realizamos una búsqueda exhaustiva en el portal “ClinicalTrials.gov” y no encontramos ningún estudio en fase IV relacionado con el desarrollo de una vacuna contra la hepatitis C. Esto sugiere que, a la fecha, no hay una vacuna aprobada en ningún país y, por lo tanto, tampoco se encontraría incluida en el calendario de vacunación de Colombia, lugar donde se documentó el caso.

A pesar de los avances en la investigación de vacunas contra la hepatitis C, se han enfrentado desafíos significativos debido a la amplia variedad de cepas del virus en todo el mundo. Se han llevado a cabo estudios de vacunación en cientos de voluntarios con un candidato de vacuna basado en la inserción de virus portadores -como el adenovirus de chimpancé (ChAd) y el virus vaccinia modificado Ankara (MVA)- con la esperanza de potenciar la respuesta inmune contra el HCV, similar a lo logrado en los estudios con animales. Sin embargo, es importante destacar que esta investigación se encuentra en la fase 1 de desarrollo y aún no se han publicado los resultados ^2^.

El *National Institute of Allergy and Infectious Diseases* (NIAID) de los Estados Unidos desarrolló un estudio de fase I-II con el propósito de evaluar la seguridad, la eficacia y la inmunogenicidad de una nueva vacuna profiláctica contra la hepatitis C. Esta vacuna se basó en el uso secuencial del virus recombinante AdCh3NSmut1 (derivado de un adenovirus no replicante) y MVA-NSm. Se incluyó un grupo placebo, y usuarios de drogas inyectables no infectados por HCV. Los resultados del estudio revelaron que la vacuna profiláctica no logró prevenir la infección crónica por HVC en este grupo de población ^3^.

Los diferentes ensayos clínicos aún no han logrado el desarrollo de una vacuna eficaz contra el virus de la hepatitis C debido a los numerosos desafíos enfrentados durante los estudios e investigaciones en este campo.

Agradecemos el conocimiento aportado por el artículo y la oportunidad de expresar nuestro comentario con el propósito de brindar información adicional relacionada con el punto mencionado.

Alessandra Basurco, Alexandra Ñato

Escuela Profesional de Medicina Humana, Universidad Privada San Juan Bautista, Lima, Perú
