# Supplementary material for: Cartas al Editor
Source: Biomedica. 2024 May 30;44(2):281–2. [Article in Spanish] doi: 10.7705/biomedica.7554 (PMC11349064; doi:10.7705/biomedica.7554)
Supplement: Related texts

# Reply: Cartas al Editor

Barraquilla, D. C., 19 de noviembre de 2023

Doctores

Alessandra Basurco y Alexandra Ñato

Universidad Privada San Juan Bautista

Lima, Perú

Estimadas doctoras:

Agradecemos a Basurco y Ñato por la carta enviada relacionada con la publicación “Colestasis intrahepática por *Treponema pallidum* en paciente inmunocompetente”.

Estamos de acuerdo con los comentarios hechos: a la fecha no hay vacuna contra la hepatitis C y solo se han hecho estudios con algunos candidatos potenciales. Nuestro texto no debió haber incluido está recomendación sobre la vacunación contra la hepatitis C.

Por otro lado, respecto a la vacunación contra la hepatitis A y B y el virus del papiloma humano, esta se debe aplicar según las indicaciones de las guías médicas locales o internacionales.

Muchas gracias

Daniela Loaiza Hernández

***
